# Cancer patients in SARS-CoV-2 infection: a single-center experience from Wuhan

**DOI:** 10.7150/jca.47065

**Published:** 2020-08-27

**Authors:** Jiafeng Wang, Jun Zhang, Yuexing Tu, Xianlong Zhou, Haijun Huang, Lina Shao, Legao Chen, Yan Zhao, Minghua Ge

**Affiliations:** 1 The 2nd Clinical Medical College, Zhejiang Chinese Medical University, Hangzhou, Zhejiang, 310053, China.; 2 Medical Aiding Team for COVID-19 in Hubei, Zhejiang Provincial People's Hospital, Hangzhou, Zhejiang, 310014, China.; 3 Hangzhou Medical School People's Hospital, Hangzhou, Zhejiang, 310014, China.; 4 Emergency Center, Zhongnan Hospital of Wuhan University, Wuhan, Hubei, 430071, China.; 5 Hubei Clinical Research Center for Emergency and Resuscitation, Zhongnan Hospital of Wuhan University, Wuhan, Hubei, 430071, China.

**Keywords:** COVID-19, Coronavirus, Pandemic, Cancer, Therapy

## Abstract

**Background:** The Coronavirus disease 2019 (COVID-19) global pandemic has posed unprecedented challenges to the health-care systems all over the world. Among the booming literatures about COVID-19, there is yet a paucity of study addressing the association between COVID-19 and cancer, which is a rare comorbidity of COVID-19, as well as consensus for treatment of cancer in this pandemic.

**Methods:** In this retrospective, single-center cohort study, information of all inpatient cases with laboratory-confirmed COVID-19 who had treatment outcome were collected from the designated departments in Zhongnan Hospital of Wuhan University, Wuhan, China on March 10, 2020. Demographic data, clinical information, and treatment outcomes were extracted from electronic medical records. Severe events were defined as admission to intensive care unit (ICU), the use of mechanical ventilation, or death.

**Result:** A total of 716 patients with laboratory-confirmed COVID-19 infection were identified. Among them, a total of 12 cases (1.7%, 95% CI: 0.7%-2.6%) had history of cancer with 4 cases (33%) experienced severe events. Compared with cases without cancer, patients with cancer have higher risks of severe events (33% vs 7.7%, *p=*0.012) and deaths (25% vs 3.6%, *p=*0.009). Multivariable logistic regression model showed that cancer was independently associated with increased odds of severe events after adjusting for other risk factors (OR 6.51, 95% CI 1.72-24.64; *p=*0.006). Among COVID-19 patients with cancer, we found that patients older than 60 years (75%), with other comorbidities (50%), or experiencing anticancer treatment in past month (42.9%) had a numerically higher incidence of severe events.

**Conclusion:** Cancer is a rare comorbidity of patients with COVID-19; however, it cannot be overemphasized due to its poorer outcomes. We propose that personalized treatment recommendation for cancer patients should be addressed during COVID-19 pandemic, along with meticulous personal protective protocols for them to mitigate the risk of SARS-CoV-2 infection.

## Introduction

In early December 2019, the first pneumonia cases of unknown origin were identified in Wuhan, the capital city of Hubei province 1. The pathogen has been identified as a novel enveloped RNA betacoronavirus 2, which has currently been named as severe acute respiratory syndrome coronavirus 2 (SARS-CoV-2) 2. The World Health Organization (WHO) declared coronavirus disease 2019 (COVID-19) to be a global pandemic on March 11, 2020. Although many COVID-19 patients have good prognosis, those patients who are in older age, or with comorbidities such as hypertension, diabetes, or coronary heart disease could result in poor prognosis 3-5. As one of the rare comorbidities, the associations between cancer and COVID-19 is not fully uncovered, as well as the treatment of cancer during this pandemic. Recently, several studies demonstrated that patients with cancer may be more susceptible to COVID-19 infection and may have poorer prognosis 6, 7. However, due to the small sample size and large heterogeneity, further studies were required to confirm their findings 8. In our present study, a total of 12 laboratory-confirmed COVID-19 patients with cancer from a single institution in Wuhan were reported. We hope that our findings of the SARS-CoV-2 associated with cancer will benefit the global community in the battle against COVID-19 infection.

## Methods

### Data source

This retrospective cohort study was approved by both the Research Ethics Commission of Zhongnan Hospital of Wuhan University (2020032) and Zhejiang Provincial People's Hospital (2020QT068). The requirement of informed consent was waived due to its retrospective design. On March 10, 2020, all medical records of inpatient cases diagnosed with COVID-19 who had treatment outcome in designated departments in Zhongnan Hospital of Wuhan University, Wuhan, China were reviewed. Demographic data, clinical information, and treatment outcome were extracted from electronic medical records. All data were collected by two of the authors listed (XLZ and YMJ) independently to ensure accuracy.

### Definitions

For this study, the severity of COVID-19 infection was defined according to the World Health Organization (WHO) interim guidance 9. Mild type infection is defined as cases where patients have non-pneumonia or mild pneumonia. Severe type infection is diagnosed when at least one of the following three diagnostic criteria is met: 1) respiratory distress (RR ≥ 30/minute); 2) resting blood oxygen saturation ≤ 93%; or, 3) arterial blood oxygen 183 partial pressure (PaO2)/FiO2 ≤ 300 mmHg. Critical type is diagnosed when at least one of the following three diagnostic criteria is met: 1) respiratory failure needing mechanical oxygenation; 2) shock; or 3) development of other organ failure, requiring intensive care unit (ICU) care. Fever was defined as axillary temperature of higher than 37.3 °C. Severe events were defined as admission to ICU, the use of mechanical ventilation, or death 6.

### Statistical Analysis

Baseline characteristics of the patients with or without cancer were shown using counts and percentages for categorical variables and medians with interquartile ranges (IQR) for continuous variables. Differences between groups were tested using the χ^2^ test or Fisher's exact test for categorical variables and t test or Wilcoxon test for continuous variables, depending on the nature of the distribution. No imputation was made for missing data. To explore factors associated with severe events, forward conditional logistic regression model was used. A two-sided α of less than 0.05 was considered statistically significant. Statistical analyses were done using R software, version3.6.2 (R Foundation for Statistical Computing).

## Results

There were a total of 775 patients with laboratory-confirmed COVID-19 infection who had treatment outcome in designated departments in Zhongnan Hospital of Wuhan University between December 30, 2019 and March 10, 2020. 59 cases (7.6%) were excluded due to unavailable of clinical data in electronic medical records. Ultimately, a total of 716 patients with laboratory-confirmed COVID-19 infection were included for further analyses. Among them, 12 cases (1.7%, 95% CI: 0.7%-2.6%) had a history of cancer, aged from 37 to 78 years (median, 57 years). Six cases of them (50%) were males. As far as type of cancer, 10 of them (83.3%) had carcinomas, one had lymphoma, and another one had leukemia. A total of 7 (58.3%) cases received anticancer treatments during the past month, which includes surgery (n=2), chemotherapy plus immunology (n=1), combined chemotherapy (n=2), single-agent chemotherapy (n=1), and radiotherapy (n=1). A total of 4 (33%) cases experienced severe events with 3 deaths (25%) occurred (**Table 1**).

Compared with patients without cancer, there is no significant difference in age, smoking history, sex, baseline symptoms, some comorbidities, and even clinical type (*p>*0.05). Intriguingly, COVID-19 patients with cancer have a significantly higher risk of severe event (33% vs 7.7%, *p=*0.012) and death (25% vs 3.6%, *p=*0.009) compared with those without cancer (**Table 2**). Multivariable logistic regression indicated that cancer was independently associated with increased odds of severe event after adjusting for other risk factors including age, gender, smoking history, and other comorbidities (OR: 6.51, 95% CI: 1.72-24.64, *p=*0.006) (**Table 3**).

Among COVID-19 patients with cancer, we found that those older than 60 years had a higher incidence of severe events than those aged 60 years or younger (75% vs 12.5%). Furthermore, the 3 patients that died were all older than 60 years. Patients with other comorbidities (50% vs 25%) or anticancer treatment in the past month (42.9% vs 20%) had higher rate of severe events than those without (**Table 4**). Among the 7 patients had anticancer therapy in the past month, three of them, two had surgery and one had chemotherapy plus immunotherapy, experienced severe events.

## Discussion

It is hypothesized patients with cancer are deemed to be at heightened risk of severe illness from COVID-19 due to their systemic immunosuppressive state caused by the malignancy and anticancer treatments 7, 10, 11. Recently, Liang W. et al. reported their finding of 18 COVID-19 patients with cancer history from a multicenter study in China, which, to the best of our knowledge, was the first to focus on COVID-19 infection in patients with cancer 6. A higher incidence of SARS-CoV-2 infection and poorer prognosis in the patients with cancer was found in their study. However, due to its small sample size and large heterogeneity, such conclusions didn't accept by other scholars 8. In our case series, there was no significant difference in age, smoking history, or some other comorbidity between patients with or without cancer. However, patients with cancer had a higher risk of severe events compared with those without cancer, which was consistent with the finding of the previous study by Liang W et al. 6. These odds were further confirmed by logistic regression after adjusting for other risk factors.

Among COVID-19 patients with cancer, we found cases with severe events were older, had more underlying comorbidities and more likely undergone anticancer therapy in past month. Older age and some comorbidities had been confirmed as well documented risk factors of both COVID-19 and cancer patients 3, 12, whereas the adverse effect of anticancer therapy which may cause immediate impairment immune function will never be overemphasized. To date, no specialized medication is available for the treatment of SARS-CoV-2 infection and the patient's immune function is a major determinant of the disease severity, thus populations with low immune function may be more vulnerable and have high mortality after COVID-19 infection. Some anticancer treatments such as surgery and chemotherapy may cause immediate impairment immune function 13, 14. In our case series, we found that all of the severe events happened in patients who received anticancer treatment in the past month, except the oldest one (78 years old) with comorbidities. Moreover, we observed of 3 severe events occurred in 7 patients with therapy in past month, 2 (including the youngest one) caused by surgery, 1 caused by combined chemotherapy plus immunotherapy, which suggested that “more aggressive” therapy would be more likely lead to severe events.

Currently, considering the infection risk and overwhelmed health-care system, postpone of anticancer therapy for stable or low-grade cancer was recommended during pandemic 6, 10. However, it remains challenging for patients with aggressive or progressive cancer, which need balance the benefits and risks of the anticancer treatment 15. Several variables, such as the extent of the epidemic, the local healthcare structure capacity, the risk of infection to the individual, the status of cancer, patients' comorbidities, and age should be considered 16. Based on the previous studies and ours, we recommend prescription of lower risk therapy, instead of aggressive therapy for controlling cancer progression, along with meticulous personal protective protocols during the special period, because “aggressive” therapy may accelerate and exacerbate disease progression once COVID-19 infection occurs. First, an intentional postponing of elective surgery should be considered. A recent study has shown that postoperative patients with confirmed COVID-19 had much higher rate of ICU admission and mortality than patients without surgery 17. Second, more active systemic therapy such as concurrent chemoradiation or chemotherapy plus immune checkpoint inhibitors (ICIs) was not recommended. Literatures indicated that ICIs could lead to higher risk rather than benefit in early period due to its unique anticancer mechanism 18, 19. Third, oral antitumor medication was recommended due to its mild side effect and no need for hospital admission. The three cases had oral antitumor medication, one from our study had capecitabine and two from previous study received target therapy, ended with good outcome. Lastly, we recommended chemotherapy as an alternative for young patients, especially for those in good overall health status. All three patients younger than 50 years who received chemotherapy in both our series and Liang W's 6 had good outcome. However, under such circumstance, toxic side effects such as myelosuppression should be closely monitored.

Several limitations associated with this study also warrant mention. First, since this study was a retrospective study, it was limited in that various biases could not be excluded. Second, we no doubt missed patients who were asymptomatic or had mild cases and who were treated at home, so our study cohort may represent the more severe end of COVID-19. Last but not least, interpretation of our findings might be limited by the small sample size with a large amount of heterogeneity.

In summary, our study demonstrated that cancer is a rare but important comorbidity of COVID-19 patients with poorer outcomes. We recommend postpone the “aggressive” therapy during the pandemic and control the aggressive or progressive cancer with low risk therapy as the first choice.

## Figures and Tables

**Table 1:** The details of 12 COVID-19 patients with cancer in Zhongnan Hospital of Wuhan University

	Age (years)	Gender	Clinical Type	Cancer Type	Anticancer treatments in the past month	Comorbidities	ICU Admission	Invasive ventilation	Death
Case 1	44	Female	Mild	Thyroid cancer	None	None	No	No	No
Case 2	64	Male	Mild	Lung cancer	None	Hypertension	No	No	No
Case 3	37	Male	Critical	Liver cancer	Surgery (Right hepatectomy)	None	Yes	No	No
Case 4	60	Male	Mild	B cell non-hodgkin's lymphoma	None	Diabetes, Mellitus; Tuberculosis	No	No	No
Case 5	46	Female	Severe	Thyroid cancer	None	None	No	No	No
Case 6	67	Female	Critical	Esophageal cancer	Surgery (Radical resection of esophageal cancer)	CHD;	Yes	Yes	Yes
Case 7	48	Female	Mild	Breast cancer	Adjuvant chemotherapy (docetaxel + pirarubicin)	None	No	No	No
Case 8	57	Male	Mild	Colon cancer	Palliative chemotherapy (capecitabine)	None	No	No	No
Case 9	57	Male	Mild	Lung cancer	Radiotherapy (mediastinum)	None	No	No	No
Case 10	61	Male	Critical	Lung cancer	Chemotherapy+ Immunotherapy (docetaxel+ cisplatinum+ camrelizumab)	None	Yes	Yes	Yes
Case 11	78	Female	Critical	Chronic lymphocytic leukemia	None	Hypertension, CHD, COPD	Yes	No*	Yes
Case 12	43	Female	Mild	Nasopharyngeal cancer	Neoadjuvant chemotherapy (docetaxel+ cisplatinum+ fluorouracil)	None	No	No	No

*The patient refused invasive ventilation. Abbreviations: ICU, Intensive care unit; CHD, Coronary heart disease; COPD, Chronic obstructive pulmonary disease.

**Table 2:** Baseline characteristics, symptoms, and outcomes of COVID-19 patients with or without cancer

Characteristics	Total	Cancer patients	Non-cancer patients	*P*-value
Age (year)	55 (42~65)	57 (44.5~63.3)	55 (40~65)	0.704
Male/Female	336(46.9%)/380 (53.1%)	6 (50%)/6 (50%)	330 (46.9%)/374 (53.1%)	0.830
Known smoking history	62 (8.7%)	2 (16.7%)	58 (8.2%)	0.340
Fever	483 (67.5%)	8 (66.7%)	475 (67.5%)	1.0
Chest discomfort	160 (22.3%)	5 (41.7%)	155 (22%)	0.204
Shortness of breath	131 (18.3%)	2 (16.7%)	129 (18.3%)	1.0
Cough	372 (52%)	5 (41.7%)	367 (52.1%)	0.472
Fatigue	236 (33%)	5 (41.7%)	231 (32.8%)	0.736
Mild/(Severe/Critical)	555 (77.5%)/161 (22.5%)	7 (58.3%)/5 (41.7%)	548 (77.8%)/156 (22.2%)	0.209
Any other comorbidity*	201 (28.2%)	4 (33.3%)	197 (28.1%)	0.940
Death	28 (3.9%)	3 (25%)	25 (3.6%)	0.009
Severe events	58 (8.1%)	4 (33.3%)	54 (7.7%)	0.012

*Other comorbidities include chronic hypertension, diabetes mellitus, obstructive pulmonary disease coronary, heart disease, and cerebrovascular disease.

**Table 3:** Logistic regression model for identifying risk factors for severe events

Variables	OR (95%CI)	*P* value
Age	1.029 (1.009-1.051)	0.005
Cancer	6.507 (1.718-24.643)	0.006
Comorbidities	3.100 (1.673-5.744)	<0.001

A forward conditional logistic model was performed. Other variables include sex and smoking. Comorbidities include: chronic hypertension; diabetes mellitus; obstructive pulmonary disease; coronary heart disease; and, cerebrovascular disease.

**Table 4:** The incidence of severe events between different groups of COVID-19 patients with cancer

Variables	Severe events (n=4)
**Age, y**	
> 60 (n=4)	3 (75%)
≤ 60 (n=8)	1 (12.5%)
**Other comorbidities**	
With (n=4)	2 (50%)
Without (n=8)	2 (25%)
**Anticancer treatment in the past month**	
Yes (n=7)	3 (42.9%)
No (n=5)	1 (20%)
**Surgery in the past month**	
Yes (n=2)	2 (100%)
No (n=10)	2 (20%)
